# A Review of Methods for Fibre-Optic Distributed Chemical Sensing

**DOI:** 10.3390/s19132876

**Published:** 2019-06-28

**Authors:** Xin Lu, Peter James Thomas, Jon Oddvar Hellevang

**Affiliations:** NORCE Norwegian Research Centre AS, P. O. Box 6031, 5892 Bergen, Norway

**Keywords:** fibre optics, fibre sensing, distributed fibre sensing, chemical sensing

## Abstract

Chemical sensing is of great importance in many application fields, such as medicine, environmental monitoring, and industrial process control. Distributed fibre-optic sensing received significant attention because of its unique feature to make spatially resolved measurements along the entire fibre. Distributed chemical sensing (DCS) is the combination of these two techniques and offers potential solutions to real-world applications that require spatially dense chemical measurements covering large length scales. This paper presents a review of the working principles, current status, and the emerging trends within DCS.

## 1. Introduction

The function of an optical fibre sensor is to measure some physical, chemical, or biological parameter that modifies some optical property of the system (e.g., intensity, polarisation, phase, etc.) [1]. Optical fibres have favourable properties such as being lightweight, small in size, low in cost, and having low attenuation and immunity to electromagnetic interference, making them an ideal sensing medium for a wide range of real-world applications [2,3]. 

Optical fibre sensors are particularly suited for chemical monitoring and analysis due to their ability to withstand harsh working conditions, and their small volume and inert nature enable them to have minimal impact on the environment. At the very beginning, fibre-optic chemical sensors exploited optical fibres just as a transmission medium to guide the light to and from the sensing region. In this type of arrangement, known as an extrinsic optical fibre sensor, the light is modified either via direct interaction with the analyte or with a sensor element outside the fibre that has some property that varies in the presence of an analyte [4]. 

For intrinsic fibre sensors, the optical fibres themselves are used as a sensing medium. For chemical sensing this is inherently difficult to achieve because the silica fibre is chemically inert. In order to overcome this, a transductive element can be used to convert the chemical information to parameters of the fibre that can be easily recovered, such as a strain/temperature or optical transmission [5,6,7,8]. For example, a Pt-loaded WO_3_/SiO_2_-coated fibre can be used for hydrogen sensing. The presence of hydrogen causes Pt/WO_3_ to release heat which can be measured using a Sagnac-loop interferometer [5]. Otherwise, at least a small portion of light must travel out of the fibre core allowing for interaction with a chemically sensitive material. Thus, a large number of fibre chemical sensors rely on microstructured optical fibres (MOF) [9] or standard fibres with structural modifications, such as fibre tapers, microfibres, tilted fibre gratings, and fibre facets [10,11,12,13]. 

An ideal chemical sensor must be very sensitive down to ppm or even ppb concentrations, be highly selective to only one particular analyte, and be totally immune to the variation of physical environmental quantities such as temperature and pressure [14]. Several fibre-optic-based chemical sensors demonstrated competitive performance in one or more of these aspects [15,16,17]. For example, the combination of surface plasmon resonance and a molecularly imprinted polymer on a plastic fibre taper demonstrated a high selectivity to l-nicotine [15]. Also, a multimode fibre tip with lutetium bisphthalocyanine could detect NO_2_ with a resolution of 0.2 ppb over a range from 0 to 5 ppm [16].

A property of fibre-optic chemical sensors is their ability to be multiplexed onto a single fibre, enabling measurements covering large scales as required by applications such as those in the oil and gas industry and for environmental monitoring [18,19,20]. Such multiplexed sensors are often also referred to as called quasi-distributed sensors since they are only capable of making measurements at predefined points, and any variation of the analyte between sensing points is simply missed. Alternatively, for fully distributed chemical sensors, the entire fibre is analyte-sensitive and, therefore, offers the possibility for spatially dense measurements. The core sensing mechanism behind any distributed chemical sensing (DCS) scheme is shared with a corresponding point fibre chemical sensing technology. In the distributed form, spatial resolvability over long distances is achieved through the application of well-established optoelectronic techniques. 

This paper presents a review of the state of the art in DCS methods and a comparative analysis of their characteristics and performance. 

## 2. Distributed Fibre Sensing

**Figure 1:**
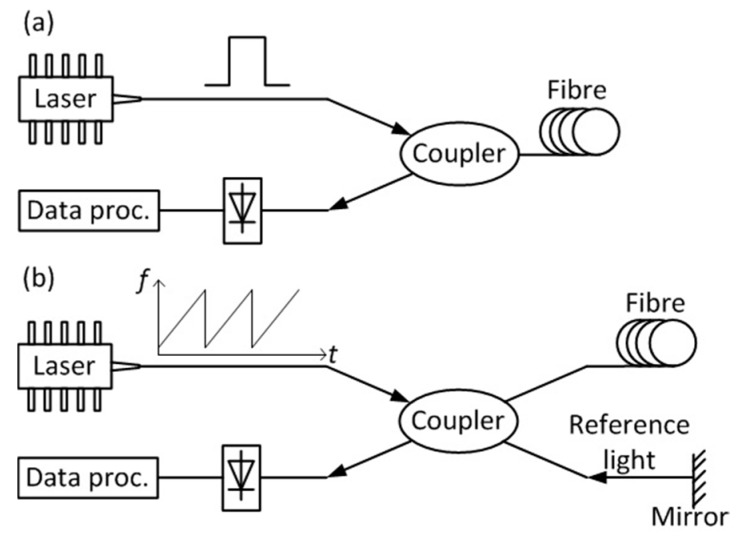
(**a**) Basic set-up of a standard optical time domain reflectometry (OTDR) system. An optical pulse is sent into the fibre and the Rayleigh backscattered light is acquired by a photodetector, then processed. (**b**) Basic set-up of a coherent optical frequency domain reflectometry (OFDR) system. The incident light is a continuous wave with frequency sweeping; the Rayleigh backscattered light from the sensing fibre is mixed with the reference light at a photodetector, and their beating is processed to retrieve the spatial information.

DCS, as a distributed fibre sensing (DFS) technique [21], is capable of employing the entire optical fibre as the sensing element and of providing measurements with a high degree of spatial density. The spatial information is usually resolved through optical time domain reflectometry (OTDR) or optical frequency domain reflectometry (OFDR). In an OTDR system, an optical pulse is launched into the fibre, and the backscattered light intensity is measured as a function of time [22], as shown in Figure 1a. The distance along the fibre to which a given backscatter component corresponds is determined by time-of-flight considerations, and the spatial resolution is commonly defined as half of the pulse length [22]. In OFDR, either the optical frequency of the light source or the frequency of an amplitude modulation is swept, for “coherent” and “incoherent” systems, respectively. The backscattered light as a function of frequency is acquired by heterodyne detection in either the optical (coherent OFDR) or electrical (incoherent OFDR) domains. The spatial information is retrieved by applying a Fourier transform to the backscatter in the frequency domain [23]. A classical coherent OFDR scheme is plotted in Figure 1b; the incident light with linearly scanning optical frequency is split by a coupler, whereby one part of the light is launched into the fibre and the other part acts as reference light. The backscattered light from the fibre is mixed with the reference light reflected by the mirror; then, their frequency beating is acquired by a photodetector. Finally, the obtained signal is processed to retrieve the spatial information. 

**Figure 2:**
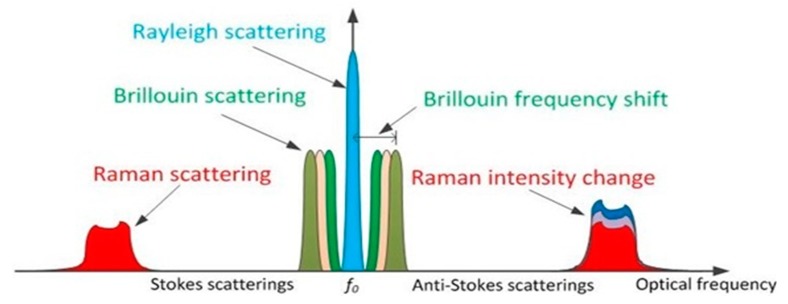
Backscattering processes used for distributed fibre sensing. *f_o_* is the frequency of the laser input. The Rayleigh scattering is due to a linear interaction of the light with the transmission medium, while all other components result from non-linear scattering processes.

The backscattered signal comprises Rayleigh, Raman, and Brillouin scattering processes inside an optical fibre, as shown in Figure 2. Different types of distributed sensor are often classified in terms of what backscattered component they are designed to measure. Rayleigh scattering is an elastic process, in which there exists no energy transfer between the incident light and the medium; thus, the backscattered light exhibits no frequency shift compared to the laser input, as illustrated in Figure 2. On the other hand, inelastic scattering, e.g., Brillouin and Raman scattering, requires an energy exchange between the light and the material; thus, the frequency of the scattered light is expected to shift from the incident light, as illustrated in Figure 2. For silica fibres with an incident light at 1550 nm, the frequency shifts of Brillouin scattering and Raman scattering are about 11 GHz and 13.2 THz, respectively [24]. So far, all the three kinds of backscattering were applied to DFS.

The classical Rayleigh-based DFS utilises a short coherence length laser source, and measures the intensity of the backscattered light, which is related to the fibre attenuation profile. The same system with a long coherence length source will cause an ordinary optical fibre to function in a way similar to an ultra-long and weak fibre Bragg grating (FBG) sensor [25], with the backscatter profile exhibiting spatially distinct features that are unique to a given optical fibre. For example, in a coherent OTDR system, the coherent length of the light source must be longer than the optical pulse width, so that the light rays backscattered within the pulse width will interfere with each other at the photodetector. Due to the interference process, environmental variations can be measured. In practice, the coherent Rayleigh backscattered light must be acquired along the fibre at different optical frequencies. Then the measurement is repeated after the environmental changes. Consequently, temperature and strain information can be obtained by cross-correlation of the coherent Rayleigh backscatter profiles [26,27,28]. This kind of sensor demonstrates a typical temperature sensitivity of ~−1.2 GHz/K and a strain sensitivity of ~−150 MHz/με. 

Spontaneous Raman scattering is a thermally activated process, where the amplitude of the anti-Stokes component is proportional to the average number of thermally generated phonons in the medium, which is expressed as

**Figure 3:**
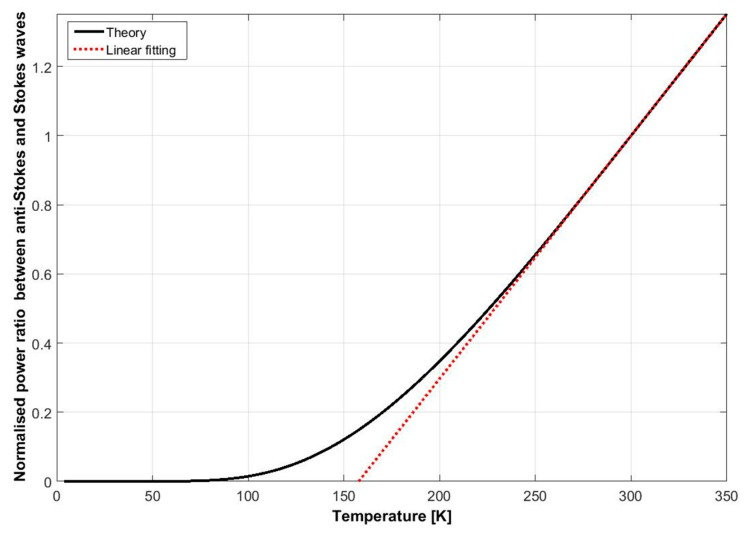
Normalised ratio between anti-Stokes and Stokes waves of Raman scattering as a function of temperature. A linear relationship between the temperature and the ratio is observed from ~250 K to 350 K.

(1) n¯=1ehΩkT−1,

where h is the Planck constant, Ω is the optical frequency shift, k is the Boltzmann constant, and T is the absolute temperature. On the other hand, the amplitude of the Stokes part is proportional to $\bar{n}+1$. Hence, Raman distributed fibre sensors can retrieve the temperature profile along the fibre based on the optical power change of the anti-Stokes component [29]. In practice, both Stokes and anti-Stokes components are usually collected and compared in order to make the measurement more robust. The ratio of the optical power between the two components is calculated and depicted as a function of temperature in Figure 3. Obviously, the ratio changes quasi-linearly with the temperature from ~200 K to 350 K. The slope represents the temperature sensitivity, which is about 0.7%/K at 300 K. However, the sensitivity vanishes quickly below 200 K because the strength of spontaneous Raman scattering is temperature-dependent as shown by Equation (1). It has to be noted that Raman sensors are sensitive only to temperature, not strain.

**Figure 4:**
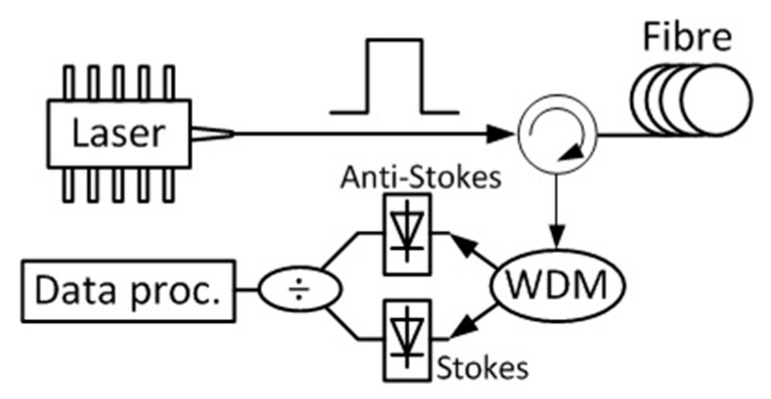
Simple scheme of a distributed Raman sensor. An intense pulse is launched into the fibre; then, the Stokes and anti-Stokes components of Raman scattering are selected by a wavelength division multiplexer (WDM) and acquired by two photodetectors. Their power ratio is obtained and processed.

A simple sensing scheme based on Raman scattering is plotted in Figure 4. An intense optical pulse is launched into the sensing fibre; then, the Stokes and anti-Stokes components of the Raman scattering are selected by a wavelength division multiplexer (WDM) and acquired by photodetectors. The ratio between the obtained signal is used to retrieve the temperate profile. Additional data processing is necessary for calibration.

**Figure 5:**
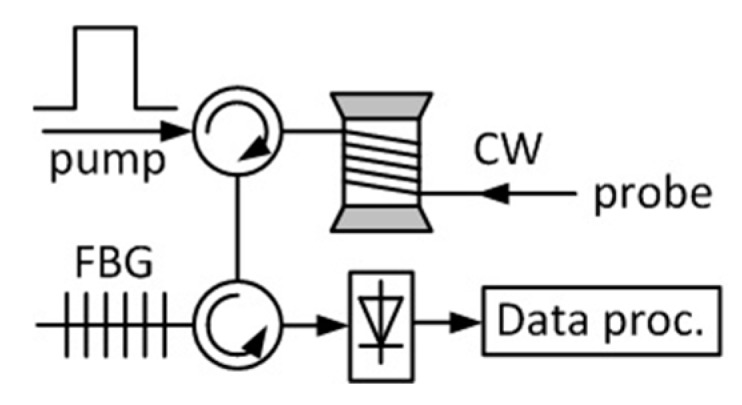
Simple scheme of a Brillouin optical time domain analyser. The pump and probe are counter-propagating in the fibre; the probe light is detected and processed to retrieve the local Brillouin frequency.

The amplitude of Brillouin scattered light is also temperature- and strain-dependent. In addition, the Brillouin frequency shift is proportional to the acoustic velocity in the medium, which is dependent on environmental conditions. Therefore, the resultant shift demonstrates a linear relationship with temperature and strain changes. Since the frequency-based measurement is inherently more robust and more stable than the intensity-based one, most current Brillouin distributed sensors measure the local Brillouin frequency to determine the environmental conditions for a better performance [30]. The basic set-up of a classical Brillouin sensor, a Brillouin optical time domain analyser, is depicted in Figure 5. An optical pulse (pump) and a probe which is a continuous wave (CW) are counter-propagating along the sensing fibre. Their optical frequency difference is scanned around the Brillouin frequency shift. At the receiver, a narrow band filter, usually an FBG, is used to select the probe and filter out the Rayleigh backscattering of the pump. The obtained Brillouin spectrum needs to be processed to find the location of the peak in order to determine the Brillouin frequency. The most popular used post-processing methods are polynomial/Lorentzian curve fitting, Lorentzian cross-correlation, and cross-reference plot analysis [31]. The Brillouin frequency, which is actually the location of the peak, is then determined with some data processing methods. The distributed Brillouin sensor is sensitive to both temperature and strain with typical sensitivities of ~1 MHz/K and 0.05 MHz/με, respectively.

The application of current DFS systems is mainly limited to fibre loss, temperature, and strain because conventional silica fibres have low sensitivity to other parameters. Advanced techniques based on Rayleigh and Brillouin scattering can also map chromatic dispersion, birefringence, and the shape of the fibre [32,33,34,35].

There are some other DFS techniques that draw less attention. White-light polarimetric sensors based on birefringent fibre work in transmission mode, and measure the polarisation coupling between the eigen modes induced by environmental perturbations [36]. The spatial information is determined by the position of peaks in the Fourier spectrum of the transmitted signal. A single long and weak FBG also acts as a distributed sensor if its reflection spectrum can be spatially resolved within the grating length [37]. In addition, different fibre interferometers were applied to distributed vibration sensing, but gave a comparatively large location error (tens of metres) [38,39,40]. These types of DFS systems are not as popular as conventional backscatter-based DFS systems due to their relative complexity and low performance. As a result, most of the less conventional forms of DFS are not applied to DCS.

In general, the performance of a DFS system can be evaluated in terms of four different parameters [3] as follows:

- Measurand resolution, which is the minimum change in measurand at a given location that the DFS is able to detect;
- Spatial resolution, which is the spatial separation over which the DFS can make independent measurements. Considering a step change in measurand, covering the entire response range of the DFS, the spatial resolution is usually defined as the distance corresponding to where the change in response is between 10 and 90% of the range of response;
- Sensing range, which is the maximum length of the fibre that can be measured with the desired performance (measurand and spatial resolution);
- Measurement time, which is the minimum time required by the sensor to achieve a measurement of a given quality.

The four parameters are interdependent with trade-offs making up the whole system’s performance. The improvement of one factor often necessitates a compromise in another. For example, OTDR systems are able to measure over 100 km with a spatial resolution of several metres. The OFDR can achieve sub-millimetre resolution, but its sensing distance is usually limited [23]. Signal-to-noise ratio (SNR) is the key parameter linking the interdependence of the four aspects. It directly determines the measurand resolution, and finally limits the spatial resolution, sensing distance, and measurement time (average times) [3].

## 3. Overview of Sensing Mechanisms for DCS

At the core of any DCS system is a mechanism for converting chemical information into some optical property modulation that can be effectively quantified using established DFS techniques. In this section, the current state of the art in distributed chemical sensors is analysed in terms of analyte measured and performance. The overall performance of a DCS technology is dependent on both the underlying chemical sensing mechanism and the optoelectronic implementation for the distributed measurement. For example, the total measurement time may be limited either by the reaction time of a chemical transducer material to a change in analyte, or the measurement time required for the optoelectronic system to achieve a given performance.

### 3.1. Optical Loss-Based Method

The simplest DCS-compatible sensing mechanism involves the conversion of chemical information into a modification of the loss properties of an optical fibre. Such properties can be easily recovered in a distributed way using techniques such as OTDR and OFDR. 

#### 3.1.1. Bending Loss

Many materials will swell owing to the absorption of other substances. Such materials can be used in a way that cause the fibre to bend as a result of the volume expansion. When the fibre is sufficiently bent, the light can be directly refracted out of the fibre core (macro-bending) or be coupled to the higher-order propagation modes (micro-bending), which experience higher loss in single-mode fibres [41]. Hence, a DCS can be realised by measuring the chemically induced fibre bending loss.

**Figure 6:**
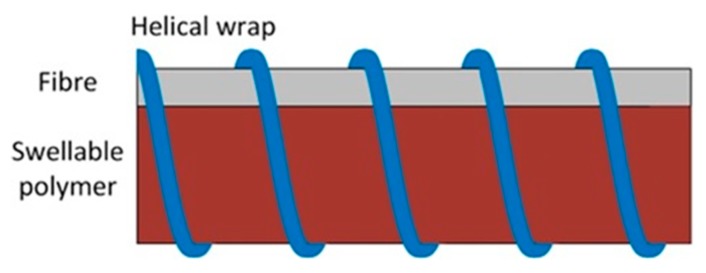
Sensing cable design for a bending loss-based distributed chemical sensor. The polymer will swell in the presence of the analyte, which then pushes the fibre to the wrap and causes fibre loss.

**Figure 7:**
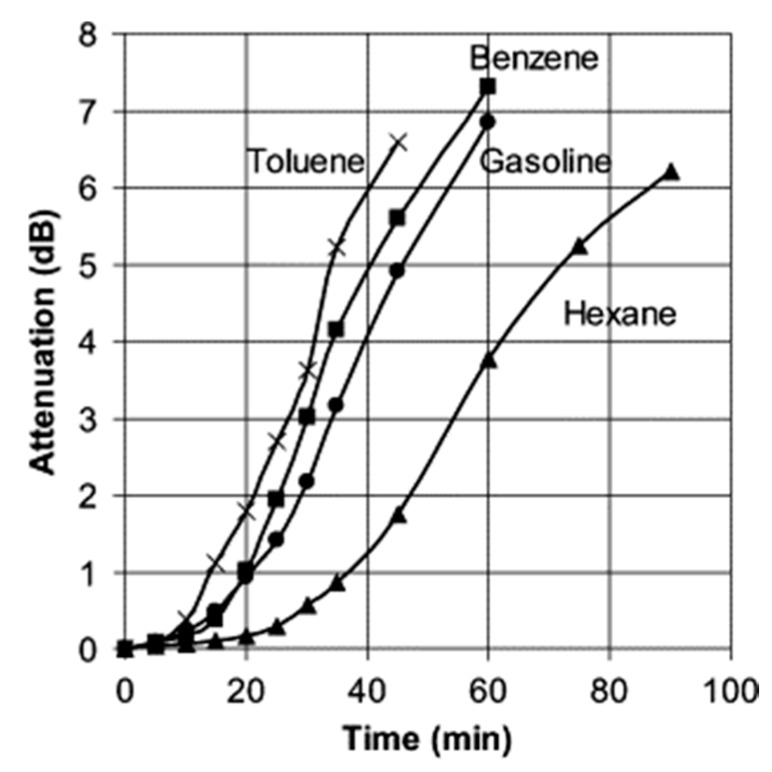
Measured bending loss when the fibre is immersed in different organic solvents. Reproduced with permission from Reference [44].

An example of a bending loss DCS sensor is shown in Figure 6, and consists of three parts: a swellable material, an optical fibre, and a helical wrap that ensures close contact between the fibre and the material. The sensitive material absorbs the analyte and expands in volume. Hence, the fibre is forced against the wrap and deformed, resulting in local bending loss which can be easily detected. To enhance the micro-bending loss, the helix period is designed as

(2) $$\Lambda =\frac{2\pi a}{\sqrt{2\Delta }},$$

where a is the core radius, and Δ is the maximum refractive index difference between fibre core and cladding [42,43]. This type of technique was used for distributed measurements of humidity, hydrocarbon liquids, and pH [42,43,44,45,46]. An example is shown in Figure 7, where large fibre attenuations are obtained by exposing the fibre to different organic solvents.

A standard optical fibre alone is subject to micro-bending due to a mechanical response of the coating when placed in a wet environment [47]. This phenomenon cannot be applied directly to humidity sensing with standard OTDR or OFDR systems because the induced loss is too weak. However, the second-order (LP11) mode experiences larger micro-bending loss compared to the fundamental mode, and distributed humidity sensing was demonstrated by measuring optical loss of the LP11 mode based on an OTDR system working at 1 μm [47].

For bending loss-based distributed chemical sensors, swellable materials with a large expansion coefficient should be used to achieve a high sensitivity. Other factors influencing choice of swelling materials are cross-sensitivity and temperature dependence [44]. 

#### 3.1.2. Absorption Loss

The identification of characteristic absorption properties of a material as a function of wavelength can be used to identify the presence of a substance [48]. While laboratory instruments normally exploit the complete spectrum in order to maximise measurement robustness, a lot of information can be gained by selecting the most relevant parts of the spectrum. For distributed measurements, the backscattered intensity profile is measured when using a transmission wavelength within the absorption spectrum of the relevant substance. A local power drop represents the presence of the sample, and the change amount is related to the concentration [49]. This method requires a direct interaction between the light and the analyte, and was performed in different types of fibres modified by tapering or etching of the cladding to ensure that a component of the evanescent wave propagates in free space. Many organic substances have absorption fingerprints in the 2–3-µm region, for which fluoride-doped fibres have high transmission. 

Microstructured optical fibres (MOF) consist of air gaps that run along the fibre, which can be filled with different analytes, presenting a good platform for chemical sensing. Hollow-core fibres are of particular interest because the light is guided in the air hole through the photonic bandgap effect [50], enabling a more efficient interaction.

**Figure 8:**
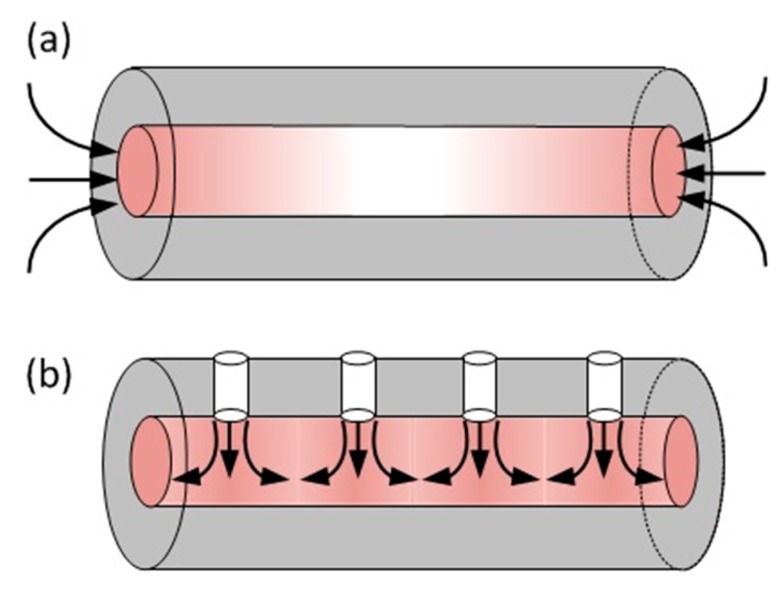
Comparison of two distributed chemical sensing schemes based on hollow-core fibres. (**a**) The analyte enters the air hole through the fibre ends; (**b**) the analyte enters the air hole through conduits along the fibre. The tint represents the concentration of the analyte.

**Figure 9:**
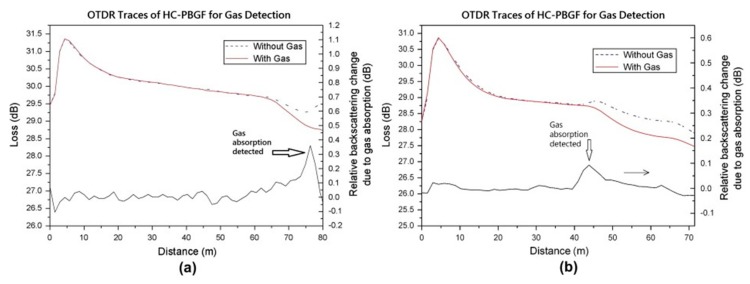
Distributed measurement of 10% acetylene based on a hollow-core fibre. (**a**) The gas was filled at the end of the fibre; (**b**) the gas was filled at 44 m of the fibre. The upper lines represent the measured OTDR traces, and the lower curves show the slope difference between the red line and dotted line. Reproduced with permission from Reference [52].

In spite of the many convincing demonstrations of discrete chemical sensing based on MOFs, this method is very difficult to implement in a distributed manner, because the analyte can enter only at the ends of the fibre. As shown in Figure 8a, the diffusion of the analyte along the fibre is non-uniform and cannot represent the local condition. Drilling conduits that connect the fibre surface to the air holes can circumvent this problem [51]. In this way, the analyte is allowed to assess the core along the fibre, making the measurement truly distributed, as shown in Figure 8b. Distributed acetylene sensing was demonstrated in a 75-m hollow-core fibre drilled with an array of such conduits [52], as shown in Figure 8. The OTDR traces from a hollow-core photonic bandgap fibre (HC-PBGF) are obtained when acetylene exists at the end and at 44 m of the fibre, as plotted in Figure 9a,b, respectively. The slope difference, shown as the lower curve, clearly exhibits the location of the acetylene.

**Figure 10:**
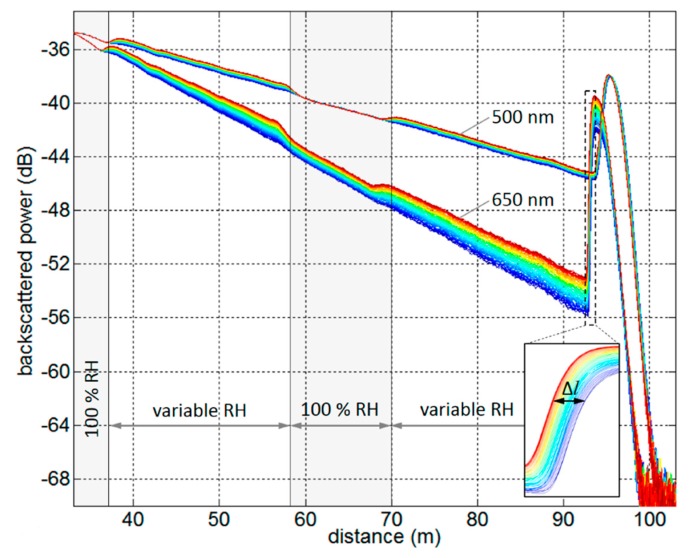
OTDR traces measured in a poly(methyl methacrylate) (PMMA) plastic optical fibre (POF) in a climate chamber with the relative humidity decreasing from 90% to 30% over five days. Reproduced with permission from Reference [55].

Plastic optical fibres (POFs) are another candidate for absorption-based DCS [53]. Some POFs are inherent humidity sensors due to the water affinity of many common polymers [54]. For example, poly(methyl methacrylate) (PMMA) can absorb ~2% water by mass, and the presence of OH bonds from water in the structure causes absorption over a broad range of wavelengths. Distributed humidity sensing was demonstrated in a PMMA POF by exploiting this OH-induced fibre attenuation as shown in Figure 10 [55]. The OTDR traces from the POF are obtained at 500 nm and 650 nm with the relative humidity decreasing from 90% to 30%.

Absorption-based DCS can also be implemented with polymer-clad silica (PCS) fibres. This kind of fibre uses a polymer, usually silicone, as the cladding material and can be easily functionalised. The analyte can diffuse into the polymer cladding and absorbs the evanescent field at the core/cladding interface [56].

Absorption loss DCS has high selectivity as long as the chosen wavelength does not coincide with absorption processes due to other species. The sensing distance is often limited by high fibre losses, and, in common with other loss-based distributed sensors, the SNR at a given position is influenced by the level of absorption loss at other positions along the fibre. In addition, laser drifts could lead to measurement errors if the drifts are not properly compensated for. 

#### 3.1.3. Guidance Loss

**Figure 11:**
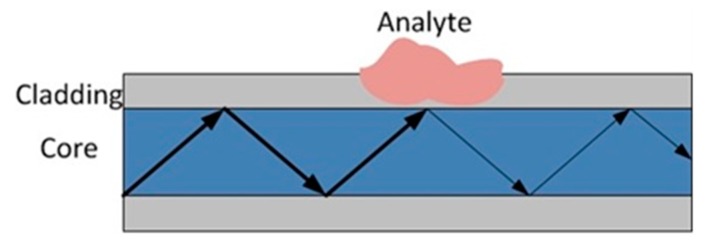
Illustration of chemical sensing based on light guidance loss. The presence of analyte in the cladding may change the guidance condition of the fibre, modifying the loss properties. The weight of the line represents the optical power.

Classical optical fibres comprise a core and a cladding with a lower refractive index (RI); thus, the light is well confined in the core by total internal reflection [57]. Chemically induced RI change in the cladding may break the light guidance condition, leading to optical power variations as shown in Figure 11. DCS based on this idea requires a cladding material that is permeable to the target analyte or reacts with the chemical. The analyte diffusion or the chemical reaction may increase the RI of the cladding, causing light leakage [58]. Alternatively, the RI of the cladding may decrease due to the analyte diffusion, turning the sensing fibre from leaky mode to waveguide mode [59]. 

Spatial variations in the concentration of a variety of substances, such as hydrocarbons and CO_2_, were spatially resolved using different PCS fibres and the guidance loss principle [58,60]. Metal cladding was also used for distributed hydrogen sensing [61], but the distance is limited due to large losses.

Since this type of sensor is sensitive to any analytes that can cause a RI change in the cladding, a special protective membrane may be necessary to exclude any interfering chemicals from interacting with the cladding.

### 3.2. Fluorescence Emission

**Figure 12:**
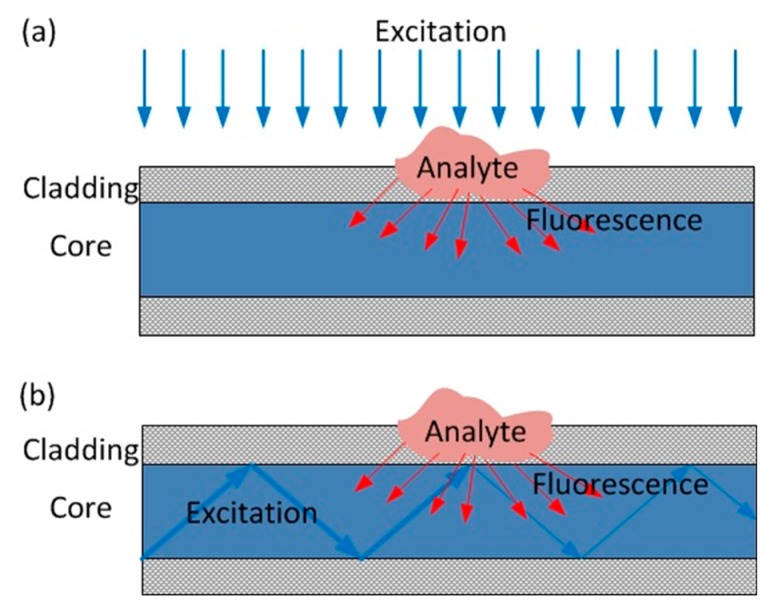
(**a**) First generation of fluorescence-based distributed chemical sensing (DCS), where the dopant is excited by side illumination. (**b**) Modified fluorescence-based DCS, where the dopant is excited by an evanescent field of the light propagating inside the core. The shaded region represents cladding immobilised with fluorophore. The weight of the line represents the optical power.

Fluorescence measurement is a very sensitive chemical sensing technique [62]. When a fluorophore interacts with an analyte, its fluorescence properties can be modified in a number of different ways including intensity, lifetime, wavelength, and/or polarisation [63]. Fluorophores were placed at the fibre tip, in fibre cladding, and even in the fibre core for point sensing [64,65,66]. For the latter approach, dopant fluorophores in the core are excited by the emission from analyte-sensitive fluorophores in the cladding [66]. Although this method enables higher fluorescence intensities in the core, doping both the cladding and core is challenging. As a result, fluorescent cladding seems to be the only practical choice for distributed fluorescence measurement. PCS fibres are mostly used for this method [67]. For distributed fluorescence measurements, OTDR is used to detect the local optical power change in the presence of the analyte. The first generation of such sensors requires an external illumination to excite the fluorescent dye in the cladding [68], as depicted in Figure 12a. This method is obviously not applicable to field applications. In later investigations, fluorophores were excited by the evanescent field of excitation light propagating inside the core, as shown in Figure 12b, making distributed sensing more practical [69]. Distributed fluorescence measurement was also demonstrated in exposed-core MOFs [70].

Distributed fluorescence measurements show high sensitivity and selectivity; thus, fluorophores with short lifetimes should be chosen to optimise spatial resolution [71]. In addition, the emission is usually in the visible range, which experiences larger fibre loss and ultimately limits the sensing distance.

### 3.3. Strain-Based Method

**Figure 13:**
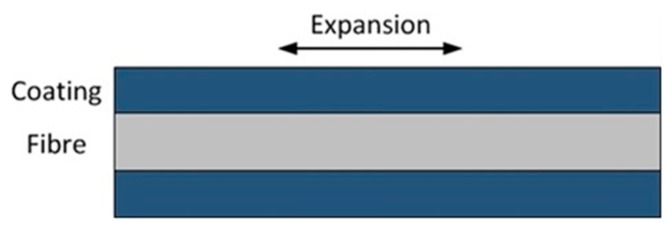
Optical fibre used for chemical sensing based on strain changes induced by a coating that expands in response to an analyte.

Chemical information can manifest itself as a strain on the fibre imparted by transducer materials that swell in response to a substance. The chemical transducer materials often take the form of a simple fibre coating, as shown in Figure 13. Polyimide and palladium (Pd) are widely used coating materials for humidity and hydrogen sensing, respectively [72,73]. The generated strain is usually acquired by measuring the wavelength shift of an FBG for discrete sensing [74].

This sensing method can be easily adapted to a fully distributed configuration, since strain can be spatially resolved by DFS based on coherent Rayleigh and Brillouin scattering. Coherent Rayleigh DFS gains more attention for this application because it is three orders of magnitude more sensitive to strain than Brillouin DFS [75]. Coherent OTDR and coherent OFDR techniques were used to demonstrate fully distributed humidity and hydrogen sensing [76,77,78]. The strain change of a polyimide-coated fibre was measured using a commercially available OFDR system, exhibiting a near-linear response to relative humidity range from 15 to 92% [78]. Brillouin-based DFS was also applied to measure the water content of clay samples as a preliminary study [79].

**Figure 14:**
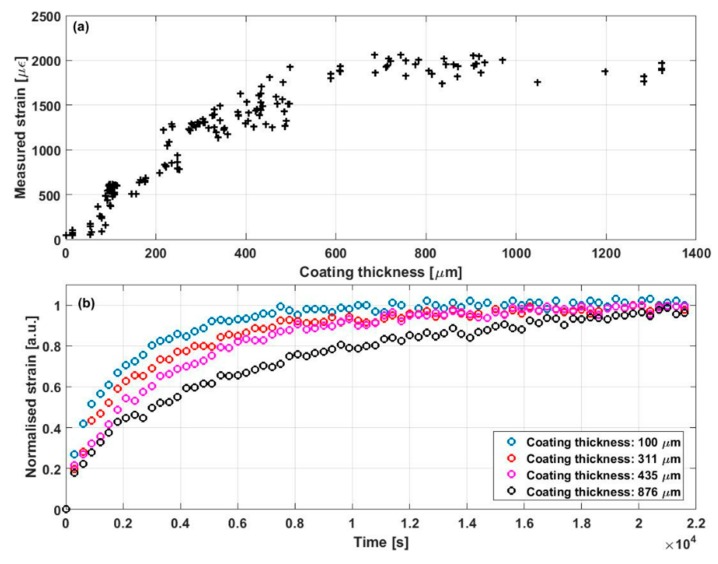
(**a**) Strain change along the test fibre as a function of coating thickness, when the relative humidity was raised from 20% to 70%. (**b**) Normalised strain response of polyimide-coated fibres with different thickness when the relative humidity changed from 15% to 30%. Modified with permission from Reference [80].

The sensitivity of such sensors is clearly dependent on the properties of the coating material, i.e., the expansion coefficient to the target analyte, the Young’s modulus, and the cross-sectional area [80]. Higher values of those parameters usually lead to an improved sensitivity. However, the increase of coating thickness results in a slower response of the sensor, simply because the analyte needs more time to diffuse in the coating. This kind of trade-off is demonstrated in Figure 14. The resultant strain is plotted in Figure 14a as a function of coating thickness under the same humidity change. Thicker coating leads to larger strain, but this impact becomes less significant as the thickness goes over 700 μm. On the other hand, Figure 14b shows the temporal evolution of humidity-induced strain measured by fibres with different coating thickness. The thicker coating (876 μm) clearly demonstrates a slower response. As a result, the coating needs to be carefully designed to find the balance between sensitivity and response time.

Since strain-based chemical sensing can be realised using optical fibres with transmission properties equal to telecommunication-grade fibre, long sensing distances are achievable. The measurement time is longer than the optical power-based method because the distributed strain measurement generally requires frequency scanning. Some approaches circumvent this problem by employing chirped pulses, where the optical frequency is modulated within the pulse duration [81,82]. Strain-based approaches are also influenced by temperature changes, and thermal expansion of the transducer introduces measurement error if it cannot be fully discriminated.

### 3.4. Temperature-Based Method

Temperature is a measurand that fibre sensors can measure directly. Temperature change is also widely observed during different chemical reactions, e.g., the photothermal effect [83] and the redox effect [5]. Many fibre temperature sensors are used to deduce the chemical information from these temperature changes, and some of them were adapted to realise DCS. 

**Figure 15:**
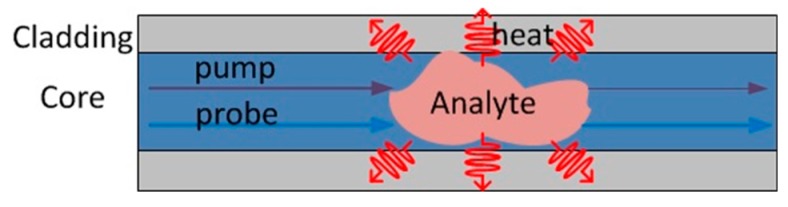
Distributed chemical sensing based on measurement of chemically generated heat. The wavelength of the pump chosen to coincide with the absorption line of the analyte. A small portion of the pump power is absorbed by the analyte and converted into heat; the probe reads the temperature change to detect the chemical. The weight of the line represents the optical power.

The absorption of optical radiation by molecules generates heat, causing variations of density, pressure, and refractive index. Gas sensing based on this idea is actually similar to the absorption-loss based measurement; pump light must be tuned to an absorption line for the analyte. Probe light at a wavelength far from an absorption feature is also necessary for reading out temperature changes triggered by the absorption. The whole process is simply illustrated in Figure 15. Garcia-Ruiz et al. gave an example of this technique being applied in a distributed way [84]. In that work, a gas sample present in the air holes of a small-core photonic crystal fibre absorbed the pump light through the evanescent field, and the small temperature increases were measured using coherent OTDR. Later, distributed gas sensing was realised in a hollow-core fibre where the gas absorption is larger. The local concentration of acetylene was retrieved by measuring the phase change experienced by the probe light due to the photothermal effect [85].

Instead of exploiting weak photothermically induced temperature changes to infer chemical information, more potent thermal perturbations can be applied using an external thermal source. This method is widely used in environmental monitoring to assess the water content in the soil [86,87,88,89]. For distributed sensing, external heat is generated by Joule heating of a metal capillary or sheath within the sensing fibre embedded within the soil. A distributed temperature sensor is used to measure the resulting temperature evolution of the soil, from which the water content can be determined given the local thermal properties such as conductivity and specific heat [86,89].

The externally induced thermal change method for inferring chemical properties involves temperature changes that are high enough to be detected by a commercial distributed temperature sensor. The sensing performance of these methods in general is affected by the ambient temperature.

### 3.5. Opto-Acoustic Coupling Method

Most of the methods mentioned above are based either on special structured fibres or standard fibres with extra transducer elements, resulting in high cost per meter of cable, which hinders these techniques from being used in real-world applications. Consequently, there was a focus toward developing DCS technologies that rely on standard telecommunication fibres. This need was answered very recently by a novel chemical sensor based on measuring the change of acoustic impedance in the medium surrounding the fibre [90]. The principle behind this is called guided acoustic wave Brillouin scattering (GAWBS) or forward stimulated Brillouin scattering [91], an opto-acoustic mechanism in which transverse acoustic modes are activated by and have influence on optical waves travelling along the fibre. The cross-section of the silica fibre acts as a resonator for the acoustic waves which are reflected at the outer surface of the cladding. The amplitude of the reflection is determined by the acoustic impedance of the silica fibre and that of the outside medium. The acoustic damping of the generated acoustic wave is measured optically and allows for the detection of any chemical species that modify the acoustic impedance. 

GAWBS opens new possibilities for chemical sensing, but its application to distributed measurements is made challenging by the fact that the excited transverse acoustic waves scatter light in the forward direction [92]. Efforts were made to realise fully distributed GAWBS measurement and resulted in the development of two distinct techniques. One of them is based on measuring the stimulated acoustic wave-mediated power coupling between two light pulses [93]. As two optical pulses co-propagate along the fibre, optical power is transferred from the higher-frequency pulse to lower-frequency one owing to the acoustic wave. The longitudinal optical power evolution is acquired by the OTDR technique. The frequency detuning between the two pulses is scanned around the excited acoustic frequency (several hundreds of MHz) to plot the GAWBS spectrum. Air, water, and ethanol were successfully identified over a 3-km fibre with 100-m spatial resolution. At the same time, a separate probing technique was proposed based on the reconstruction of local GAWBS spectrum by retrieving the phase evolution of the guided light modulated by the stimulated acoustic wave [94]. Using this method, a 15-m spatial resolution over 730 m was achieved. The spatial resolution in both sensors is mainly limited by the system noise.

This distributed application of GAWBS for chemical sensing is definitely in its infancy; thus, the performance is not comparable with some mature techniques. For example, the analyte concentration can only be determined at a percentage level even for discrete sensing [90]. However, this idea attracts enormous interest due to the unique feature of employing unmodified, standard fibre, and it is developing very fast since the first demonstration. For example, bare fibres without any coating were used at the beginning; however, different experiments were successfully demonstrated using polyimide-coated fibres [93,95,96]. It is consequently realistic to expect improved GAWBS probing schemes in the near future that will allow better performance in distributed chemical sensing

### 3.6. Other Methods

There are other DCS methods based on some unpopular or complicated DFS techniques; thus, they draw less attention. Distributed salinity sensing was demonstrated in a polyimide-coated polarisation-maintaining (PM) photonic crystal fibre [97]. The swelling of the coating modifies the fibre birefringence, which is measured by a Brillouin dynamic grating. However, the generation of such a grating requires high-power optical beams with precise frequency and polarisation adjustment [98].

The white-light polarimetric sensor was also applied to DCS [99]. An air hole along the sensing PM fibre is necessary for the access of the analyte, just like the air hole in the MOF for chemical sensing. The presence of the sample in the hole induces polarisation coupling, and the displacement of the liquid droplet in the air hole was successfully measured. However, this is essentially a distributed refractive index sensor; thus, its selectivity is low.

## 4. Discussion

All the DCS methods presented above involve largely differing measurement principles and exhibit different performances. Their relative performance must be evaluated according to their merits as chemical sensors and their applicability to distributed measurements. Ideally, a wide range of evaluation metrics as possible should be considered. However, in many cases, these evaluation metrics are interdependent. As explained above, the sensitivity can be improved by increasing the thickness of the reactive fibre coating, but the response time becomes longer. The practical complexity of the DCS system is also a key parameter of suitability for most applications.

Table 1 provides an overview of DCS performance based on the analysis in the last section. The measurand resolution is proportional to the sensitivity for a given SNR; thus, both parameters are represented by the latter in the table. A quantitate comparison of different DCS technologies is challenging because, to the best of our knowledge, no single publication provides all the relevant information required for such an analysis. Since all the parameters in Table 1 are interlocked, only a complete list of all the related values allows for a fair comparison of the different sensing methods. In order to promote the development of this field, it is suggested here that all the parameters need to be addressed when presenting DCS technologies in future publications.

Many DCS sensors have the potential for meter-scale spatial resolutions over multi-kilometre ranges. The measurement range is assumed to be only fibre loss-dependent, determined by the sensing fibre. As an example, Table 2 lists the loss properties of different fibres used in several reported DCS applications. Both spatial resolution and range are dependent on the chosen DFS technique. The limiting factor determining the measurement time, on the other hand, is most often the rate of the chemical interaction between the analyte and transducer material. The measurement time ranges from seconds to minutes, depending on the required sensitivity or analyte resolution.

The optical power variation-based DCS measurements are the simplest in terms of optical interrogation hardware since standard OTDR systems can be used. The distributed temperature and strain-based DCS sensors are comparatively complex. The optoacoustic coupling DCS measurement is the most complicated because it needs to excite the acoustic wave and reconstruct the GAWBS spectrum. However, this technique employs standard fibres as the sensing medium, whereas other DCS methods require special sensing fibres; in particular, the bending loss-based sensor needs specially engineered sensing cables, as shown in Figure 6. 

The fluorescence-based method demonstrates good sensitivity and selectivity. It is most often performed using PCS fibres, but POFs with doped cladding [100] can be used as well. Distributed chemical sensors using these fibres could be applied to other sensing mechanisms, e.g., absorption and guidance loss [101]. Therefore, the sensitivity and selectivity of methods based on absorption and wave guidance loss are also marked as high. Sensitivity at ppm or sub-ppm level was achieved using PCS fibres, dependent on the exposed fibre length and measurement time [102]. This method is a promising candidate for chemical analysis over length scales of a few kilometres.

The DCS methods based on strain and temperature demonstrate sensitivity at percentage concentration levels, which is comparatively lower than the fluorescence-based methods; however, they can easily reach tens of kilometres in range. They are, thus, suitable for large-area applications with a lower requirement on sensitivity, such as environmental monitoring and leakage detection. However, special measures may be necessary to improve their selectivity.

Generally speaking, all DCS methods are temperature-dependent. DCS based on different loss mechanisms suffers measurement error due to other factors, e.g., power variation of the laser and extra loss due to environmental perturbations. In addition, the chemically induced loss at a given location leads to a lower SNR for the rest of the fibre, affecting the analyte resolution, which might eventually limit the sensing range.

The methods based on bending loss and opto-acoustic coupling seem to have relatively unfavourable characteristics overall. Bending loss-based sensors, represent some of the earliest demonstrations of DCS, but exhibit lower performance than other methods that were developed subsequently. Opto-acoustic coupling-based DCS represents one of the most recent developments, and deserves more attention due to its unique feature, whereby only the standard fibre is needed. Better performance can be expected after a short period of development.

It has to be pointed out that excellent repeatability and long-term stability are important prerequisites for DCS in real applications. While the information available in the literature is limited regarding medium- and long-term performance of DCS technologies, changes in the optical and material properties of the sensor can potentially lead to instability. For example, the ageing of the chemical transducer materials used in the bending loss-based, temperature-based, and strain-based methods may change the sensing response. Similarly, dyes used in fluorescence-based DCS may leach from the host material or bleach over time. DCS systems based on unmodified standard optical fibres alone or in combination with robust coatings are perhaps most suited to long-term applications.

## 5. Conclusions

This paper presents a comprehensive overview of distributed fibre-optic chemical sensors presented in the scientific literature. The essential information on working principles and performance are provided. The characteristics, performance, and trade-offs between different chemical sensing technologies are discussed. Until now, DCS proved useful in smart sensing applications such as hydrogen, hydrocarbon, and humidity measurements. More distributed chemical sensors with better performance, and those that measure other chemical parameters can be expected given the wide range of potential real-world applications, and the extensive research and development efforts worldwide.

## Figures and Tables

**Table 1:** Performance evaluation for different distributed chemical sensors.

Type	Bending-Loss	Absorption	Guidance	Fluorescence	Strain	Temperature	Optoacoustic
Sensitivity	Low	High	High	High	Medium	Medium	Medium
Selectivity	Low	High	High	High	Low	Low	Medium
Immunity to environmental perturbations	Low	Low	Low	Medium	Medium	Medium	Medium
Spatial resolution	Metres	≤1 m	≤1 m	Metres	≤1 m	≤1 m	Tens of metres
Sensing range	Kilometres	≤1 km	Kilometres	<1 km	Kilometres	Kilometres	Kilometres
Measurement time	Minutes	<1 min	<1 min	<1 min	<1 min	<1 min	<1 min
Sensing fibre simplicity	Low	Medium	Medium	Medium	Medium	Medium	High
Readout simplicity	High	High	High	High	Medium	Medium	Low

**Table 2:** Fibre loss for different reported distributed chemical sensors. PMMA—poly(methyl methacrylate); HC-PBGF—hollow-core photonic bandgap fibre.

Type	Used Fibre	Typical Fibre Loss	Reference
Bending loss	Graded-index multi-mode fibre	4 dB/km @ 850 nm	[43]
Absorption	HC-PBGF fibre PMMA polymer optical fibre	<30 dB/km @ 1550 nm 90 dB/km @ 500 nm	[51] [54]
Guidance	Polymer-clad silica fibre	10.6 dB/km @ 850 nm	[57]
Fluorescence	Polymer-clad silica fibre	0.29 dB/m @ 430 nm	[67]
Strain	Verrillon VHS100 series fibre	<0.6 dB/km @ 1550 nm	[77]
Temperature	HC-PBGF fibre Leoni 50/125 multimode fibre	<30 dB/km @ 1550 nm <0.6 dB/km @ 1310 nm	[84] [88]
Optoacoustic	Standard single-mode fibre	0.2 dB/km @ 1550 nm	[92,93]
